# The Limits of Pairwise Correlation to Model the Joint Entropy. Comment on Nguyen Thi Thanh et al. Entropy Correlation and Its Impacts on Data Aggregation in a Wireless Sensor Network. *Sensors* 2018, *18*, 3118

**DOI:** 10.3390/s21113700

**Published:** 2021-05-26

**Authors:** Benoît Legat, Luc Rocher

**Affiliations:** 1 Information and Communication Technologies, Electronics and Applied Mathematics (ICTEAM), Université Catholique de Louvain, B-1348 Louvain-la-Neuve, Belgium; benoit.legat@uclouvain.be; 2 Department of Computing, Imperial College London, London SW7 2AZ, UK; 3 Data Science Institute, Imperial College London, London SW7 2AZ, UK

Information theory is a unifying mathematical theory to measure information content, which is key for research in cryptography, statistical physics, and quantum computing [1,2,3]. A central property of information theory is the entropy, a metric quantifying the amount of information encoded in a signal [4]. In “Entropy Correlation and Its Impacts on Data Aggregation in a Wireless Sensor Network”, Nga et al. propose a general entropy correlation model to study the dependence patterns between multiple spatio-temporal signals [5]. They derive lower and upper bounds on the overall information entropy from only marginal and pairwise entropies, and use these bounds to study the impact of correlation on data aggregation, compression, and clustering of signals. Replicating these findings, however, we show that these bounds were incorrect, over- and underestimating the actual association patterns depending on the data. Deriving constraints and bounds on joint entropies is still a computationally difficult task and an active field of research [1,6], and new inequalities are regularly found [7,8,9,10,11]. More work is likely to be needed in order to develop a simple and general entropy correlation model for spatio-temporal signals.

Nga et al. study a system of *m* random variables $X_{1},X_{2},\ldots ,X_{m}$. They propose a normalized measure of correlation between two variables *Y* and *Z*, defined as:

(1) $$\rho \left(Y,Z\right)=2-2\frac{H(Y,Z)}{H(Y)+H(Z)}$$

with *H* the Shannon entropy [4]. The authors further denote by $\rho_{\min}=\min_{i\neq j}\rho \left(X_{i},X_{j}\right)$ and $\rho_{\max}=\max_{i\neq j}\rho \left(X_{i},X_{j}\right)$ the minimum and maximum correlation between pairs of variables; $H_{\min}=\min_{i}H\left(X_{i}\right)$ and $H_{\max}=\max_{i}H\left(X_{i}\right)$ the minimum and maximum individual entropies.

The general entropy correlation model proposed by the authors rely on two claims, both incorrect:

**Claim** **1.** *In Equation (13) and Section 2.2.2, Nga et al. claim that higher-order correlations are bounded by pairwise correlations:*

$$\forall \left(i,j,k\right),\;\rho_{\min}\leq \rho \left(X_{ij},X_{k}\right)\leq \rho_{\max}$$

**Claim** **2.** *In Equations (16) and (20), Nga et al. use Claim 1 to prove that, for any subset of m variable, its joint entropy $H_{m}$ is bounded by:*

$$l_{m}H_{\min}\leq H_{m}\leq k_{m}H_{\max}$$

*with $l_{m}=\frac{2-\rho_{\max}}{2}\left(l_{m-1}+1\right)$, $k_{m}=\frac{2-\rho_{\min}}{2}\left(k_{m-1}+1\right)$, and $l_{1}=k_{1}=1$.*

We propose two examples for n=3, demonstrating that all four inequalities are incorrect. In our first example, we obtain $\rho_{\min}>\rho \left(X_{ij},X_{k}\right)$ which contradicts the lower bound of Claim 1 and $H_{3}>k_{3}H_{\max}$, which contradicts the upper bound of Claim 2.

**Proposition** **1.** *Consider the four i.i.d. discrete random variables $Y_{1},Y_{2},Y_{3},Z$ uniformly distributed over {0,1}. For the random variables ${\left(X_{i}\right)}_{i=1}^{3}=\left(Y_{i},Z\right)$, we have $\rho_{\min}=1/2$, $\rho (X_{ij},X_{k})=2/5$ for any permutation (i,j,k) of (1,2,3), $k_{3}=15/8$, $H_{3}=4$ and $H_{\max}=2$.*

**Proof.**  As $Y_{1},Y_{2},Y_{3},Z$ are independent, we have $H\left(X_{i}\right)=H\left(Y_{i}\right)+H\left(Z\right)=2$ for i=1,2,3 and $H\left(X_{ij}\right)=H\left(Y_{i}\right)+H\left(Y_{j}\right)+H\left(Z\right)=3$ for i≠j. Using Equation (1), we have $\rho (X_{i},X_{j})=1/2$ for i≠j hence $\rho_{\min}=1/2$, $k_{2}=2-\rho_{\min}=3/2$ and $k_{3}=\left(k_{2}+1\right)k_{2}/2=15/8$. For any permutation (i,j,k) of (1,2,3), we have $H_{3}=H\left(X_{ijk}\right)=H\left(Y_{i}\right)+H\left(Y_{j}\right)+H\left(Y_{k}\right)+H\left(Z\right)=4$ hence $\rho (X_{ij},X_{k})=2/5$. □

In our second example, we obtain $\rho_{\max}<\rho \left(X_{ij},X_{k}\right)$, which contradicts the upper bound of Claim 1 and $H_{3}<l_{3}H_{\min}$, which contradicts the lower bound of Claim 2.

**Proposition** **2.** *Consider three discrete random variables $X_{1},X_{2},X_{3}$ uniformly distributed over {0,1} that are pairwise independent and satisfying the equation $X_{1}⊕X_{2}⊕X_{3}=0$ where ⊕ denotes the xor operation. We have $\rho_{\max}=0$, $\rho (X_{ij},X_{k})=2/3$ for any permutation (i,j,k) of (1,2,3), $l_{3}=3$, $H_{3}=2$ and $H_{\min}=1$.*

**Proof.**  We have $H(X_{i})=1$ for i=1,2,3 and as the variables are pairwise independent, $H\left(X_{ij}\right)=H\left(X_{i}\right)+H\left(X_{j}\right)=2$ for i≠j. Using Equation (1), we have $\rho (X_{i},X_{j})=0$ for i≠j hence $\rho_{\max}=0$, $l_{2}=2-\rho_{\max}=2$ and $l_{3}=\left(l_{2}+1\right)l_{2}/2=3$. For any permutation (i,j,k) of (1,2,3), we have $H_{3}=H\left(X_{ijk}\right)=2$ hence $\rho (X_{ij},X_{k})=2/3$. □

Overall, the two new inequalities derived by Nga et al. for the joint entropy $H_{m}$ do not appear to be correct starting at m=3. The errors in the model stem from the assumption made in Claim 1 that pairwise and higher-order associations share the same minimum and maximum. The authors validate their method on a very specific dataset with $\rho_{\min}=0.6$, $H_{\min}=2.16$, and $H_{\max}=2.55$, yet our examples show that different association structures yield widely different joint entropies. Bounding the joint entropy allows the authors to study the impact of correlation on data aggregation, compression, and clustering of signals. Although different bounds could potentially offer similar results, the broader conclusions of this article may not hold in practice.

Finally, deriving constraints and bounds on joint entropies is a computationally difficult task and an active field of research [1,6,7,8,9,10,11]. Theoretical derivations and numerical estimations both have to be used to bound the joint entropy $H_{m}$, based upon research on entropic vectors. The entropic vector of the random variables $X_{1},X_{2},\ldots ,X_{m}$ is the vector of the entropies of all $2^{m-1}$ subsets of these variables. The set of all entropic vectors is a convex cone, for which a polyhedral outer-approximation is known (Theorem 1, [12]). For instance, we derive below tight (the tightness is a consequence of the fact that Equations (2) and (3) completely describe the entropic cone (Theorem 2, [12])) lower and upper bounds for $H_{3}$ in Proposition 3, suggesting an alternative approach that could lead to upper bounds for n>3 and lower bounds as well. This bound relies on the following inequalities (Theorem 2.34, [6]):

(2) $$H\left(X_{I}\right)\leq H\left(X_{J}\right)$$

which is valid for any subsets I⊆J⊆{1,…,m} and

(3) $$H\left(X_{I}\right)+H\left(X_{J}\right)\geq H\left(X_{I\cap J}\right)+H\left(X_{I\cup J}\right)$$

which is valid for any subsets I,J⊆{1,…,m}.

**Proposition** **3.** *For any three random variables $X_{1},X_{2},X_{3}$, the following inequalities hold:*

$$\begin{array}{c} \max\left(H\left(X_{12}\right),H\left(X_{23}\right),H\left(X_{31}\right)\right)\leq H_{3}\leq \min(H\left(X_{31}\right)+H\left(X_{12}\right)-H\left(X_{1}\right),\\ \;H\left(X_{12}\right)+H\left(X_{23}\right)-H\left(X_{2}\right),H\left(X_{23}\right)+H\left(X_{31}\right)-H\left(X_{3}\right)). \end{array}$$

**Proof.**  For any permutation (i,j,k) of (1,2,3), by Equation (2) with I={i,j} and J={i,j,k}, we have $H\left(X_{ij}\right)\leq H\left(X_{ijk}\right)=H_{3}$ and by Equation (3) with I={i,j} and J={j,k}, we have $H\left(X_{ij}\right)+H\left(X_{jk}\right)\geq H\left(X_{ijk}\right)+H\left(X_{j}\right)$, which implies that $H_{3}=H\left(X_{ijk}\right)\leq H\left(X_{ij}\right)+H\left(X_{jk}\right)-H\left(X_{j}\right)$. □

Similar bounds can be obtained for m>3 using Equations (2) and (3) but their tightness is not guaranteed as the entropic cone is not completely described by these inequalities for m>3 (Theorem 6, [13]). This gap could be reduced numerically by iteratively producing linear cuts, in order to refine the polyhedral outer-approximation of the entropic cone given by Equations (2) and (3) [14]. Taken together, our findings suggest that theoretical derivations (m≤3) and numerical approximations (m>3) on the entropic cone might provide future research directions towards a robust general entropy correlation model.
